# Pregnancy loss and risk of incident CVD within 5 years: Findings from the Women's Health Initiative

**DOI:** 10.3389/fcvm.2023.1108286

**Published:** 2023-02-21

**Authors:** Catherine E. Wright, Daniel A. Enquobahrie, Sarah Prager, Ian Painter, Charles Kooperberg, Robert A. Wild, Ki Park, Shawnita Sealy-Jefferson, Mary A. Kernic

**Affiliations:** ^1^Department of Epidemiology, University of Washington, Seattle, WA, United States; ^2^Department of Health Systems and Population Health, University of Washington, Seattle, WA, United States; ^3^Department of Obstetrics and Gynecology, University of Washington School of Medicine, Seattle, WA, United States; ^4^Washington State Department of Health, Olympia, WA, United States; ^5^Department of Biostatistics, University of Washington, Seattle, WA, United States; ^6^Fred Hutchinson Cancer Center, Seattle, WA, United States; ^7^Department of Obstetrics and Gynecology, Oklahoma University Health Sciences Center, Oklahoma City, OK, United States; ^8^Department of Epidemiology and Biostatistics, Oklahoma University Health Sciences Center, Oklahoma City, OK, United States; ^9^Division of Cardiovascular Medicine, University of Florida College of Medicine, Gainesville, FL, United States; ^10^College of Public Health, Division of Epidemiology, The Ohio State University, Columbus, OH, United States

**Keywords:** cardiovascular disease, pregnancy loss, miscarriage, stillbirth, epidemiology

## Abstract

**Background:**

Previous studies have demonstrated an increased risk of cardiovascular disease (CVD) in women with a history of pregnancy loss. Less is known about whether pregnancy loss is associated with age at the onset of CVD, but this is a question of interest, as a demonstrated association of pregnancy loss with early-onset CVD may provide clues to the biological basis of the association, as well as having implications for clinical care. We conducted an age-stratified analysis of pregnancy loss history and incident CVD in a large cohort of postmenopausal women aged 50–79 years old.

**Methods:**

Associations between a history of pregnancy loss and incident CVD were examined among participants in the Women's Health Initiative Observational Study. Exposures were any history of pregnancy loss (miscarriage and/or stillbirth), recurrent (2+) loss, and a history of stillbirth. Logistic regression analyses were used to examine associations between pregnancy loss and incident CVD within 5 years of study entry in three age strata (50–59, 69–69, and 70–79). Outcomes of interest were total CVD, coronary heart disease (CHD), congestive heart failure, and stroke. To assess the risk of early onset CVD, Cox proportional hazard regression was used to examine incident CVD before the age of 60 in a subset of subjects aged 50–59 at study entry.

**Results:**

After adjustment for cardiovascular risk factors, a history of stillbirth was associated with an elevated risk of all cardiovascular outcomes in the study cohort within 5 years of study entry. Interactions between age and pregnancy loss exposures were not significant for any cardiovascular outcome; however, age-stratified analyses demonstrated an association between a history of stillbirth and risk of incident CVD within 5 years in all age groups, with the highest point estimate seen in women aged 50–59 (OR 1.99; 95% CI, 1.16–3.43). Additionally, stillbirth was associated with incident CHD among women aged 50–59 (OR 3.12; 95% CI, 1.33–7.29) and 60–69 (OR 2.06; 95% CI, 1.24–3.43) and with incident heart failure and stroke among women aged 70–79. Among women aged 50–59 with a history of stillbirth, a non-significantly elevated hazard ratio was observed for heart failure before the age of 60 (HR 2.93, 95% CI, 0.96–6.64).

**Conclusions:**

History of stillbirth was strongly associated with a risk of cardiovascular outcomes within 5 years of baseline in a cohort of postmenopausal women aged 50–79. History of pregnancy loss, and of stillbirth in particular, might be a clinically useful marker of cardiovascular disease risk in women.

## Introduction

Approximately one out of four clinically recognized pregnancies ends in pregnancy loss; rates of subclinical pregnancy loss are far higher (1). Among women with a history of pregnancy loss, an increased risk of cardiovascular disease (CVD) has been observed (2–6); rates are even higher among women with a history of recurrent pregnancy loss (RPL) and stillbirth (2, 5–9). In a meta-analysis conducted by Oliver-Williams et al., a history of pregnancy loss was associated with 45% greater odds of developing coronary heart disease (CHD), while RPL was associated with nearly twofold greater odds (10). More recently, Parker et al. found odds ratios of 1.19 (95% CI 1.08–1.32), 1.18 (95% 1.04–1.34), and 1.27(95% CI 1.07–1.51) for CHD among women with a history of one miscarriage, two or more miscarriages, and any history of stillbirth, respectively, among participants in the Women's Health Initiative (WHI), a large-scale prospective study of postmenopausal women (5).

The association between pregnancy loss and age at CVD onset is less well understood. Previous research has shown a stronger association between pregnancy loss and heart disease in very young women (<age 35) than older women (9); however, less is known about the relative strength of the association among women in midlife (aged 50–59) compared with older age. Whether a history of pregnancy loss is associated with an increased risk of early-onset CVD (before the age of 60) is a question of interest and might provide clues about the biological basis for the association. Although all factors underlying the association between pregnancy loss and CVD risk are not understood, a genetic basis has been suggested (11). As the contribution of genetic factors to disease risk appears to be particularly strong for early-onset disease (12), a demonstrated association between pregnancy loss and early-onset CVD might be indirect evidence in support of the postulated genetic basis.

Additionally, such findings might inform the clinical management of women with a history of pregnancy loss. It has been proposed that, in addition to conventional cardiovascular risk factors, such as dyslipidemia, diabetes, and hypertension, the inclusion of reproductive factors into cardiovascular risk profiles might aid clinicians in identifying patients who would benefit from monitoring and control of cardiovascular disease risk factors (13). In particular, the addition of reproductive factors into cardiovascular risk profiles might be most beneficial for predicting the risk of heart disease in younger women, prior to the onset of conventional cardiovascular risk factors (14).

In the current study, we sought to expand upon the work of Parker et al. (5) by assessing whether the association between a history of pregnancy loss and CVD risk in postmenopausal women differs across age strata; in particular, whether the association is strongest among women under the age of 60. We conducted all-ages and age-stratified analyses of the associations between pregnancy loss and risk of total CVD and three major types of CVD (CHD, congestive heart failure, and stroke) within 5 years of baseline among WHI participants.

## Methods

### Study setting and study population

The WHI cohort has previously been described in detail (15). Briefly, WHI is a large-scale prospective study of postmenopausal women, aged 50–79 at baseline, who were enrolled at 40 clinical centers throughout the United States between 1993 and 1998. The main WHI study concluded in 2005; follow-up of surviving participants is ongoing in WHI Extension Studies (16). The WHI study involves both an observational study (OS) arm and three overlapping randomized trials. The latter comprise a hormone replacement therapy clinical trial (CT) for the prevention of CHD, and two studies of non-hormone treatment: dietary modification for the prevention of breast and colorectal cancer, and calcium/vitamin D supplementation for hip fracture prevention (17).

Participants who were screened for the CT but were either ineligible or unwilling to undergo randomization were invited to participate in the OS (18), a prospective longitudinal study comprising a periodic collection of data on participant demographic and lifestyle factors and health outcomes. The OS focuses on identifying novel risk factors and biomarkers of disease; primary outcomes of interest are CHD, stroke, breast and colorectal cancer, fracture, and mortality (17). In total, 161,808 participants enrolled in the WHI; the OS cohort comprised 93,676 participants (18, 19). At baseline, 31.7, 44.0, and 24.3% of the cohort were aged 50–59, 60–69, and 70–79, respectively (17).

**Figure 1:**
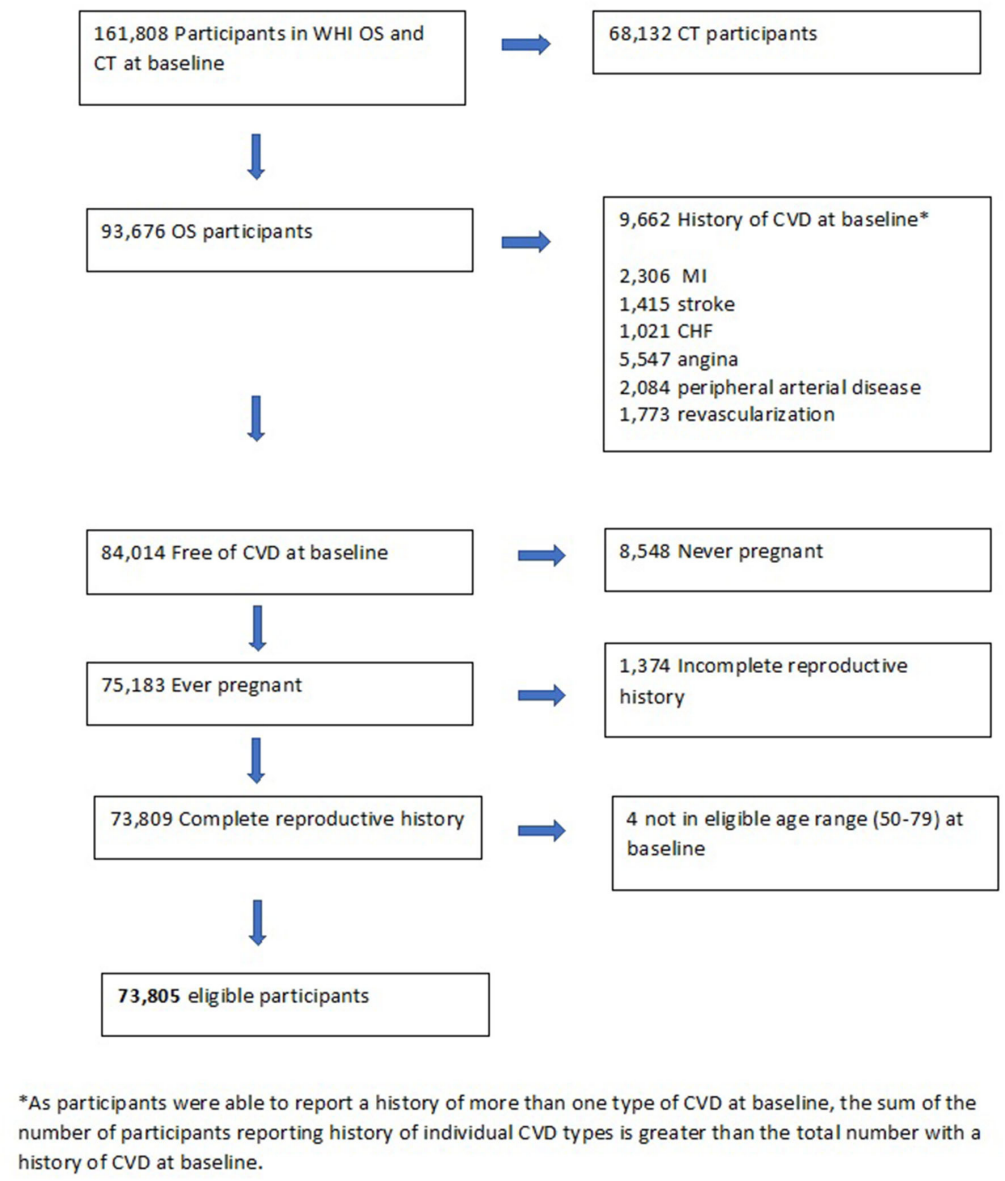
Identification of WHI participants eligible for inclusion in the current study.

The current analysis was limited to OS participants to exclude the potential effects of CT interventions on CVD outcomes. Participants eligible for inclusion were those who had ever been pregnant, for whom complete reproductive history information was available and who were free of cardiovascular disease at baseline. Of the 93,676 OS participants, 73,805 (78.8%) met the inclusion criteria (Figure 1).

### Exposure assessment

Reproductive history data were collected at the second WHI screening visit by questionnaire (14). Pregnancy history data included self-reported gravidity, parity, number of live births, spontaneous miscarriages, and stillbirth following a pregnancy lasting at least 6 months. Exposures considered in the current analysis were (1) any history of pregnancy loss (defined as at least one miscarriage and/or stillbirth), (2) history of RPL (defined as a history of two or more miscarriages and/or stillbirths), (20) and (3) any history of stillbirth.

### Outcomes assessment

The ascertainment and adjudication of primary and secondary outcomes for WHI have been described in detail previously (21). In brief, OS study participants were contacted by mail annually to collect self-reported outcomes, as well as updated exposure data (15). The adjudication of outcomes for all OS participants continued through August 2009, allowing for an average duration of follow-up for OS participants of 12 years (17). The initial adjudication of outcomes was performed by a physician adjudicator at a local clinical center and consisted of a physician review of hospital discharge summaries, relevant diagnostic tests, and death certificates. Primary and safety outcomes were subsequently confirmed by central adjudication; a review of primary cardiovascular outcomes was performed by the WHI Cardiovascular Central Adjudication Committee (21).

Outcomes for the current analysis were adjudicated total CVD (fatal and non-fatal) and three major types of CVD: CHD, heart failure, and stroke, occurring within 5 years of baseline. These comprised primary (CHD) or secondary (CVD, heart failure, and stroke) cardiovascular outcomes in the WHI CT and were also ascertained among OS participants; (21) methods for ascertainment of these outcomes were therefore well documented and consistent across local clinical centers.

Cardiovascular outcomes were defined as in the WHI OS. Non-fatal CVD outcomes were defined as CHD, stroke, heart failure, peripheral vascular disease, angina, coronary artery bypass graft (CABG), coronary revascularization, and pulmonary embolism (21). Fatal CVD outcomes were defined as death due to cerebrovascular, definite CHD, possible CHD, pulmonary embolism, other cardiovascular, or unknown cardiovascular causes.

The outcome of CHD in WHI OS participants was defined as hospitalized myocardial infarction (MI) (definite or probable) or coronary death (21). Definite and probable MI events were identified by an algorithm comprising medical history data, electrocardiogram readings, and cardiac enzyme/troponin levels, as available (22). Silent MI events were not ascertained in OS participants; therefore, silent MI was not considered as an outcome in this analysis. Fatal coronary outcomes comprised out-of-hospital as well as hospitalized deaths: coronary death was identified based on a physician review of medical records and death certificate data and was defined as death consistent with an underlying cause of death of CHD (21).

Outcome of heart failure was defined as signs and symptoms of heart failure together with one of the following: pulmonary edema on X-ray; ventricular dilation/poor ventricular function; or physician diagnosis and treatment for heart failure. Stroke was defined as rupture or obstruction of the brain arterial system, resulting in rapid neurological deficit persisting for 24 h or more. Stroke outcome comprised stroke, hemorrhagic stroke, or cause of death reported as stroke. Heart failure and stroke not resulting in hospitalization were not considered as WHI outcomes (21).

### CVD risk factor assessment

Physical measurements, blood specimens, and an inventory of current medication/supplement use were collected from OS participants during a baseline clinic visit. Participants also completed questionnaires covering medical history, family history, reproductive history, lifestyle, and behavioral factors (15).

### Confounding variables

Multivariable models were adjusted for socioeconomic factors, CVD risk factors, and other covariates identified *a priori* as potential confounders. Socioeconomic factors included in the models were education level (<high school education, high school graduate, some college/associate's degree, college graduate) and neighborhood socioeconomic status (NSES) quartile; the latter is a composite measure based on census tract-level neighborhood variables (23). Other covariates included were number of pregnancies (continuous), smoking status (never, former, or current smoker), race/ethnicity (American Indian/Alaskan Native, Asian/Pacific Islander, Black/African American, Hispanic/Latino, non-Hispanic White, or Other), aspirin use (yes/no), and body mass index (BMI) at baseline (continuous).

### Data analyses

Demographic and reproductive history were compared for three exposure categories: any history of pregnancy loss, a history of RPL, and any history of stillbirth. Multivariable logistic regression analyses were performed to assess associations between pregnancy loss and incident outcome events within 5 years of study entry. To finely adjust for age, analyses were conducted for each 1-year interval of age at study entry. The results of logistic regression analyses for each 1-year age interval were in turn used to assess associations in the entire study sample and to conduct an age-stratified analysis across three age strata: 50–59, 60–69, and 70–79 at baseline.

Multivariable logistic regression models were adjusted for socioeconomic factors, CVD risk factors, and other covariates identified *a priori* as potential confounders, as described above. Odds ratios for calculated outcomes were calculated for each of the three age strata. As the outcome of total CVD comprises the individual outcomes of CHD, heart failure, and stroke, the analyses were considered to involve three rather than four separate outcomes; thus, adjustment for multiple comparisons was not performed.

To assess the significance of associations between age and pregnancy loss exposures, regression analyses including a term for the interaction between age at baseline and history of pregnancy loss were performed for the full study sample for each outcome. A likelihood ratio test was used to compare models including a term for the interaction between age and pregnancy loss exposure to models with no interaction term.

To examine associations between a history of pregnancy loss and early-onset CVD, Cox regression analyses were used to assess incident CVD, CHD, heart failure, and stroke occurring before the age of 60 in the subset of study participants aged 50–59 at baseline. Cox regression analyses were adjusted for the same set of *a priori* established confounders included in the logistic regression analyses.

All analyses were performed using Stata 16.0 (24).

## Results

### Study sample

Table 1 shows descriptive statistics for the study sample across the three exposure categories (any history of pregnancy loss [miscarriage and/or stillbirth], a history of RPL [two or more miscarriages and/or stillbirths], and a history of stillbirth). A history of any pregnancy loss was reported by 33.8% of study subjects; histories of RPL and stillbirth were reported by 11.9 and 4.1% of subjects, respectively.

**Table 1:** Baseline demographic characteristics, cardiovascular disease risk factors, and reproductive history in selected participants from the Women's Health Initiative Observational Study (WHI OS).

	**No history of pregnancy loss *N* = 44,840**	**Any history of pregnancy loss (miscarriage or stillbirth) *N* = 24,965**	**No history of recurrent (2+) pregnancy loss *N* = 65,009**	**History of recurrent pregnancy loss *N* = 8,796**	**No history of stillbirth *N* = 70,751**	**History of stillbirth *N* = 3,054**
**Demographics**	*n* (%)	*n* (%)	*n* (%)	*n* (%)	*n* (%)	*n* (%)
**Age group**						
50–59	16,794 (34.4)	7,671 (30.7)^a^	2,986 (33.8)	2,479 (28.2)^a^	23,615 (33.4)	850 (27.8)^a^
60–69	21,392 (43.8)	11,476 (46.0)^a^	28,681 (44.1)	4,187 (47.6)^a^	31,440 (44.4)	1,428 (48.6)^c^
70–79	10,654 (21.8)	5,818 (23.3)^a^	14,342 (22.1)	2,130 (24.2)	15,696 (22.2)	776 (25.4)^a^
**Race/ethnicity**						
American Indian/Alaskan Native	187 (0.4)	121 (0.5)^c^	266 (0.4)	42 (0.5)	289 (0.4)	19 (0.6)
Asian/Pacific Islander	1,501 (3.1)	633 (2.5)^a^	1.954 (3.0)	180 (2.1)^a^	2,051 (2.9)	83 (2.7)
Black/African American	3,290 (6.8)	2,282 (9.2)^a^	4,606 (7.1)	966 (11.0)^a^	5,149 (7.3)	423 (13.9)^a^
Hispanic/Latino	1,678 (3.5)	1,056 (4.2)^a^	2,269 (3.5)	465 (5.3)^a^	2,492 (3.5)	242 (8.0)^a^
White, non-Hispanic	41,501 (85.2)	20,522 (82.4)^a^	55,036 (84.9)	6,996 (79.8)^a^	59,802 (84.8)	2,230 (73.3)^a^
Other/unknown	541 (1.1)	280 (1.1)	705 (1.1)	116 (1.3)^c^	776 (1.1)	45 (1.5)
**Education**						
<HS diploma	2,180 (4.5)	1,310 (5.3)^a^	2,920 (4.5)	570 (6.5)^a^	3,206 (4.6)	284 (9.4)^a^
High school	8,163 (16.84)	3,970 (16.0)^b^	10,769 (16.7)	1,364 (15.6)^c^	11,630 (16.6)	503 (16.6)
Some college/ associate's degree	17,563 (36.2)	9,542 (38.5)^a^	23,589 (36.6)	3,516 (40.3)^a^	25,910 (36.9)	1,195 (39.5)^b^
College graduate	20,558 (42.4)	9,938 (40.1)^a^	27,220 (42.2)	3,276 (37.5)^a^	29,452 (42.0)	1,044 (34.5)^a^
**Neighborhood socioeconomic status (NSES) quartile**						
1 (lowest)	10,598 (24.2)	5,944 (26.6)^a^	14,251 (24.4)	2,291 (29.2)^a^	15,616 (24.6)	926 (33.8)^a^
2	10.942 (25.0)	5,599 (25.0)	14,613 (25.1)	1,928 (24.6)	15,872 (25.0)	669 (24.4)
3	11,110 (25.4)	5,438 (24.3)^b^	14,663 (25.1)	1,885 (24.0)^c^	15,950 (25.2)	598 (21.8)^a^
4 (highest)	11,141 (25.4)	5,394 (24.1)^a^	14,796 (25.4)	1.739 (22.2)^a^	15,990 (25.2)	545 (19.9)^a^
**CVD risk factors**						
**Body mass index category**						
Underweight (< 18.5)	545 (1.1)	263 (1.1)	723 (1.1)	85 (1.0)	754 (1.1)	30 (1.0)
Normal (18.5–24.9)	20,054 (41.6)	9,532 (38.6)^a^	26,467 (41.2)	3,119 (36.0)^a^	28,376 (40.4)	973 (31.5)^a^
Overweight (25.0–29.9)	16,520 (34.2)	8,421 (34.1)	21,977 (34.2)	2,964 (34.2)	23,890 (34.2)	1,051 (34.9)
Obese (>=30)	11,138 (23.1)	6,467 (26.2)^a^	15,097 (23.5)	2,508 (28.9)^a^	16,653 (23.8)	952 (31.6)^a^
**Aspirin use**						
Yes	8,629 (17.7)	4,498 (18.0)	11,546 (17.8)	1,581 (18.0)	12,605 (17.8)	522 (17.1)
No	40,211 (82.3)	20,467 (82.0)	52,463 (82.2)	7,215 (82.0)	58,146 (82.2)	2,532 (82.9)
**Smoking status**						
Never smoked	25,086 (52.0)	12,238 (49.6)^a^	33.099 (51.5)	4,225 (48.7)	35,784 (51.2)	1,540 (51.2)
Former smoker	20,441 (42.4)	10,731 (43.5)^b^	27,344 (42.6)	3,828 (44.2)^b^	29,922 (42.8)	1,250 (41.6)
Current smoker	2,733 (5.7)	1,698 (6.9)^a^	3,807 (5.9)	615 (7.1)^a^	4,204 (6.0)	218 (7.2)^b^
**Reproductive history**						
**Number of pregnancies**						
1	5,469 (11.2)	621 (2.5)^a^	6,077 (9.4)	0 (0.0)^a^	6,027 (8.5)	63 (2.1)^a^
2	15,530 (31.8)	1,667 (6.7)^a^	16,912 (26.0)	285 (3.2)^a^	16,969 (24.0)	228 (7.5)^a^
3 to 4	21,781 (44.6)	10,809 (43.3)^b^	30,399 (46.8)	2,191 (24.9)^a^	31,443 (44.4)	1,147 (37.6)^a^
5 or more	6,060 (12.4)	11,868 (47.5)^a^	11,621 (17.9)	6,307 (71.7)^a^	16,312 (23.1)	1,616 (52.9)^a^
**Number of live births**						
0	1,195 (2.5)	1,168 (4.7)^a^	1,907 (2.9)	456 (5.2)^a^	2,240 (3.2)	123 (4.0)^b^
1	5,478 (11.2)	2,259 (9.1)^a^	6,891 (10.6)	846 (9.6)^b^	7,397 (10.5)	340 (11.1)
2	15,984 (32.7)	6,393 (25.6)^a^	20,398 (31.4)	1,979 (22.5)^a^	21,640 (30.6)	737 (24.1)^a^
3 to 4	20,852 (42.7)	10,740 (43.1)	27,964 (43.0)	3,638 (41.4)^b^	30,311 (42.8)	1,291 (42.3)
5 or more	5,331 (10.9)	4,395 (17.6)^a^	7,849 (12.1)	1,877 (21.3)^a^	9,163 (13.0)	563 (18.4)^a^

a *p* < 0.001 for exposed vs. unexposed subjects.

b *p* < 0.01 for exposed vs. unexposed subjects.

c *p* < 0.05 for exposed vs. unexposed subjects.

Several demographic and lifestyle factors differed across the categories of pregnancy loss. Compared with women with no history of pregnancy loss, a higher percentage of women with any history of pregnancy loss were aged 60–69 or 70–79 at baseline, identified as Black/African American or Hispanic/Latino, had either less than a high school education or had completed some college or an associate's degree, were in the lowest quartile of NSES, were obese, and were former or current smokers. Additionally, significant differences in reproductive histories were reported: women with any history of pregnancy loss were more likely than those with no history of loss to report five or more total pregnancies and either no live births or several (five or more) live births.

Differences between women with and without a history of RPL and stillbirth were nearly identical to those observed between those with and without any history of pregnancy loss. A higher percentage of women with a history of either RPL or stillbirth were aged 60–69 at baseline compared with women with no history of those exposures; the percentage of women with a history of stillbirth who were aged 70–79 at baseline was also higher than those with no history of stillbirth. Additionally, women with a history of either RPL or stillbirth were more likely to identify as Black/African American or Hispanic/Latino, have less than a high school education or have completed some college or an associate's degree, be in the lowest NSES quartile, be obese, and be current smokers; women with a history of RPL were also more likely to be former smokers than those with no history of RPL. Women reporting a history of either RPL or stillbirth were more likely than women without the respective exposures to report five or more total pregnancies and either no live births or several live births (five or more).

### Logistic regression analysis, all age groups

Within 5 years of baseline, 2,735 (3.71%) study subjects experienced CVD events; incident CHD, heart failure, and stroke occurred in 756 (1.02%), 641 (0.87%), and 724 (0.98%) subjects, respectively. After adjustment for confounders, a history of any pregnancy loss was significantly associated with incident CHD (OR 1.29 [1.08, 1.54]) 5 years post-baseline, while a history of RPL was associated with both incident CVD (OR 1.17 [1.03, 1.34]) and heart failure (OR 1.35 [1.03, 1.76]). A history of stillbirth was significantly associated with all CVD outcomes, with adjusted ORs of 1.47 (1.22, 1.75), 1.81 (1.31, 2.50), 1.76 (1.26, 2.45), and 1.53 (1.08, 2.16) for incident CVD, CHD, heart failure, and stroke, respectively (Table 2).

**Table 2:** Odds of incident cardiovascular disease (CVD) within 5 years of baseline in WHI participants aged 50–79 with and without a history of pregnancy loss, recurrent pregnancy loss, and stillbirth.

**Exposure**	Adjusted odds ratio (95% CI)^*^			
	**Total CVD**	**CHD**	**Heart failure**	**Stroke**
History of pregnancy loss	1.09 (0.99, 1.20)	**1.29 (1.08, 1.54)**	1.10 (0.90, 1.35)	1.03 (0.86, 1.25)
History of recurrent (2+) pregnancy loss	**1.17 (1.03, 1.34)**	1.16 (0.90, 1.49)	**1.35 (1.03, 1.76)**	1.15 (0.90, 1.49)
History of stillbirth	**1.47 (1.22, 1.75)**	**1.81 (1.31, 2.50)**	**1.76 (1.26, 2.45)**	**1.53 (1.08, 2.16)**

* Adjusted for age (meta-analysis of 1-year age intervals), education, NSES, number of pregnancies, smoking status, race/ethnicity, aspirin use, and BMI. CIs are not corrected for multiple comparisons. Bold values indicate a statistically significant association.

### Logistic regression analysis, age-stratified

Interaction terms between age and pregnancy loss exposures were not significant for any cardiovascular outcome (Supplemental Table 1). Therefore, results of the age-stratified analysis are provided simply to augment the all-ages analysis (Table 3).

**Table 3:** Odds of incident cardiovascular disease (CVD) within 5 years in women with and without a history of pregnancy loss, recurrent pregnancy loss, and stillbirth, by age at study baseline.

**Exposure**	**Age group**	Adjusted odds ratio (95% CI)^*^			
		**Total CVD**	**CHD**	**Heart failure**	**Stroke**
Any pregnancy loss	50–59	1.19 (0.89, 1.59)	1.45 (0.85, 2.46)	1.18 (0.63, 2.22)	0.86 (0.44, 1.69)
	60–69	1.08 (0.94, 1.25)	1.20 (0.90, 1.59)	1.10 (0.81, 1.49)	1.18 (0.88, 1.58)
	70–79	1.07 (0.93, 1.24)	**1.34 (1.03, 1.73)**	1.09 (0.81, 1.46)	0.95 (0.73, 1.24)
Recurrent (2+) loss	50–59	1.25 (0.82, 1.89)	0.75 (0.32, 1.77)	**2.18 (1.001, 4.76)**	**2.60 (1.10, 6.16)**
	60–69	**1.33 (1.10, 1.62)**	1.16 (0.78, 1.71)	**1.54 (1.03, 2.31)**	1.12 (0.75, 1.68)
	70–79	1.00 (0.82, 1.23)	1.25 (0.88, 1.78)	1.04 (0.70, 1.55)	0.90 (0.62, 1.30)
Stillbirth	50–59	**1.99 (1.16, 3.43)**	**3.12 (1.33, 7.29)**	2.45 (0.96, 6.22)	0.97 (0.23, 4.06)
	60–69	**1.46 (1.11, 1.92)**	**2.06 (1.24, 3.43)**	1.65 (0.97, 2.79)	1.32 (0.75, 2.31)
	70–79	**1.37 (1.05, 1.78)**	1.35 (0.84, 2.19)	**1.69 (1.04, 2.76)**	**1.77 (1.11, 2.80)**

* Adjusted for age (meta-analysis of 1-year age intervals), education, NSES, number of pregnancies, smoking status, race/ethnicity, aspirin use, and BMI. CIs are not corrected for multiple comparisons. Bold values indicate a statistically significant association.

After adjustment for confounders, a history of any pregnancy loss was not associated with greater odds of incident CVD 5 years post-baseline in any age group, while RPL was associated with increased odds of CVD among women aged 60–69 at baseline. A history of stillbirth was associated with incident CVD within 5 years among all age groups, with adjusted ORs of 1.99 (1.16, 3.43), 1.46 (1.11, 1.92), and 1.37 (1.05, 1.78) among women aged 50–59, 60–69, and 70–79 at baseline, respectively.

A history of any pregnancy loss was associated with incident CHD among women aged 70–79 at baseline (OR 1.34 [1.03, 1.73]). Stillbirth was associated with incident CHD among women aged 50–59 and 60–69 at baseline, with adjusted ORs of 3.12 (1.33, 7.29) and 2.06 (1.24, 3.43), respectively.

A history of RPL was associated with heart failure among women aged 50–59 (OR 2.18 [1.001, 4.76]) and 60–69 (OR 1.54 [1.03, 2.31]) at baseline. A history of stillbirth was associated with heart failure among women aged 70–79 at baseline (OR 1.69 [1.04, 2.76]); marginally insignificant adjusted ORs of 2.45 (0.96, 6.22) and 1.65 (0.97. 2.79) were observed among women aged 50–59 and 60–69, respectively.

A history of RPL was associated with stroke among women aged 50–59 at baseline (OR 2.60 [1.10, 6.16]). A history of stillbirth was associated with stroke among women aged 70–79 at baseline (OR 1.77 [1.11, 2.80]).

### Survival analysis, early-onset cardiovascular outcomes

In the subset of 24,465 study participants aged 50–59 at baseline, the rate of incident CVD before the age of 60 was 1.95 events per 1,000 person-years; rates of incident CHD, heart failure, and stroke before the age of 60 were 0.64, 0.49, and 0.20 per 1,000 person-years, respectively. (Supplemental Table 2). After adjustment for confounders, Cox proportional hazard regression analyses did not demonstrate significantly increased hazard ratios for cardiovascular outcomes before the age of 60 (Supplemental Table 3), although a marginally non-significantly elevated hazard ratio for heart failure (2.53 [0.96, 6.65]) was observed among women with a history of stillbirth.

## Discussion

In a large cohort of postmenopausal women aged 50–79, after adjustment for cardiovascular risk factors, a history of stillbirth was found to be associated with all cardiovascular outcomes within 5 years of study entry. Although we did not observe an interaction between age and pregnancy loss exposures for cardiovascular outcomes, we conducted an age-stratified analysis to determine whether meaningful patterns emerged. In the age-stratified analysis, the strongest association between a history of stillbirth and total incident CVD was observed among women aged 50–59, with smaller but still significant associations observed among women aged 60–69 and 70–79, although overlapping confidence intervals were observed for all age groups. Additionally, stillbirth was associated with incident CHD within 5 years among women aged 50–59 and 60–69. Among women aged 50–59 with a history of stillbirth, the risk estimate for heart failure was elevated but marginally insignificant after adjustment for confounders (*p* = 0.06), possibly due to the relatively small number of cases of heart failure occurring within 5 years of baseline in this age group (*n* = 70).

In a subset of study participants aged 50–59 at baseline, the proportion of study subjects experiencing cardiovascular outcomes before the age of 60 was small, and pregnancy loss exposures were not associated with significant increases in hazard ratios for any cardiovascular outcome. However, as with the age-stratified analysis, the hazard ratio for heart failure before the age of 60 was non-significantly (*p* = 0.06) elevated among women with a history of stillbirth.

The results of our analysis contribute to the existing literature demonstrating an increased risk of CVD among women with a history of pregnancy loss (2–9), and in particular a history of stillbirth (5, 9). Further, although we did not observe a significant interaction between age and pregnancy loss exposures in our analyses, our findings of strong associations between a history of stillbirth and incident CVD and CHD in women aged 50–59 augment the existing literature. The question of whether a history of pregnancy loss is more predictive of CVD risk in young women has been previously considered; however, previous work has examined CVD risk in very young women (under the age of 35) compared with older women. In a population-based study comprising more than 1 million women, Ranthe et al. found that in women under the age of 35, the rates of MI, cerebral infarction, and renovascular hypertension increased by 35–55% with each documented miscarriage. In women aged 35 and over, rates of the above outcomes increased by 6–7% per additional miscarriage (9). As the analysis of Ranthe et al. used dichotomized age groups of <35 and 35 and older, their study did not address the question of whether the association between pregnancy loss and risk of heart disease is stronger for women in midlife compared with older age.

In our all-ages analysis, our findings of higher odds of CVD, CHD, and heart failure among women with a history of stillbirth compared with any history of pregnancy loss (miscarriage or stillbirth) are consistent with previous research (5, 9, 25) and demonstrate biological plausibility. While numerous factors—including maternal diabetes, hypertensive disorders, other chronic diseases, maternal infection, and fetal genetic abnormalities—are known to increase the risk of pregnancy loss throughout gestation (26), the etiology of pregnancy loss also differs markedly by gestational age. Chromosomal abnormalities are likely to lead to losses early in pregnancy, while factors associated with loss in mid-to-late pregnancy include antiphospholipid syndrome, cervical weakness, anomalies of the uterus, infection, and placental insufficiency (27, 28). As stillbirth is more likely to occur because of maternal health factors than miscarriage, particularly miscarriage occurring in early pregnancy, stillbirth may also be more reflective of women's cardiovascular risk.

The stronger association observed between stillbirth and CVD may also provide clues about the biological basis for this association. In particular, the role that underlying vascular pathology and endothelial dysfunction (the failure of the epithelial cells to regulate homeostasis in the vascular system) (29) may play in the observed association between pregnancy loss and CVD risk is of interest. Endothelial dysfunction has been shown to be associated with adverse pregnancy outcomes (30), is an early marker of atherosclerosis (31), and is thought to play a role in the pathogenesis of heart failure (32). It has been postulated that endothelial dysfunction resulting in poor placentation during a woman's reproductive years and the development of CVD later in life might underlie the observed associations between adverse pregnancy outcomes and CVD risk (28).

As a larger proportion of stillbirths than miscarriages are attributable to placental factors, such a mechanism would be consistent with the results of our analysis. Furthermore, unlike miscarriages occurring because of fetal chromosomal abnormalities, which are primarily due to *de novo* errors of meiosis and are unlikely to recur (27), vascular pathology is likely to lead to recurrent loss (9). This would be consistent with the findings of previous research in this area showing that the risk of CVD increases with the number of previous pregnancy losses, (5, 9) and also with the increased odds of CVD and heart failure observed in the current study among women with a history of recurrent loss.

The results of our analysis complement previous research suggesting that the inclusion of a history of pregnancy loss—in particular, a history of stillbirth—into cardiovascular risk profiles might improve risk prediction. In their study of CVD among WHI participants with a history of pregnancy loss, Parker et al. noted that a history of pregnancy loss might prove to be a clinically useful marker of future CVD risk in women (5). Even in studies finding a high prevalence of the conventional risk factors of smoking, hypertension, diabetes, and dyslipidemia among patients with CVD, approximately 15% of women experiencing CVD events had none of these factors (33). Further, the positive predictive value of conventional factors has been called into question, as a high percentage of patients who do not experience CVD events also demonstrate one or more of these factors (34).

The 2011 revision of the American Heart Association's (AHA) ‘Evidence-Based Guidelines for Cardiovascular Disease Prevention in Women’ called for complications of pregnancy to be considered in evaluating a woman's lifetime risk of CVD (35). Noting that complications of pregnancy may be seen as a “failed stress test” (35), whereby pre-existing vascular or metabolic disease or endothelial dysfunction are revealed (13), the guidelines recommend postpartum follow-up to monitor and control cardiovascular risk factors for women experiencing pregnancy-related complications. However, the 2011 AHA guidelines cite only a history of pre-eclampsia, gestational diabetes, or pregnancy-induced hypertension as major cardiovascular risk factors; no mention is made of pregnancy loss.

Similarly, the 2018 “Guideline on the Management of Blood Cholesterol” from the American College of Cardiology (ACC) states that, in order to assess atherosclerotic CVD risk in women, clinicians should obtain “a thorough pregnancy-related history”0.3 (36) Examples given of pregnancy-associated disorders associated with increased risk of atherosclerotic CVD include hypertension in pregnancy, pre-eclampsia, gestational diabetes, and delivery of a low-birthweight or preterm infant; as in the 2011 AHA guidelines, pregnancy loss is not explicitly mentioned.

A 2017 editorial on pregnancy-related events and CVD risk assessment recommended that clinicians include the following in women's cardiovascular risk profiles: a history of preterm delivery, pre-eclampsia, gestational hypertension, gestational diabetes, and infant size (13). While the editorial does note recent research showing an association between pregnancy loss and CVD in later life, the authors state only that “the mechanism remains uncertain” and stop short of explicitly calling for the consideration of pregnancy loss to be regarded as a risk factor for CVD. More recently, a position paper from the European Society of Cardiology explicitly mentioned the apparent association between recurrent pregnancy loss and CVD risk, in support of a statement that pregnancy history is integral to cardiovascular risk assessment in women (37).

In a recent analysis using data from the WHI OS, Parikh et al. considered the contribution of reproductive risk factors to CHD risk prediction. In a model adjusted for conventional CHD risk factors, a history of miscarriage and stillbirth were associated with a risk of incident CHD. The inclusion of reproductive factors resulted in a modest improvement in model discrimination, although net reclassification of risk category (<5%, 5–10%, or >10% 10-year risk of CHD) for women with CHD events was not significantly improved (*p* = 0.18) (14). The authors postulated that the inclusion of reproductive factors into risk profiles might be most efficacious in predicting CHD risk in younger women, prior to the onset of the conventional risk factors of dyslipidemia, diabetes, and hypertension.

The current analysis, like that of Parikh et al., comprised postmenopausal women; thus, we cannot draw from our results any conclusions about the strength of the association between pregnancy loss and cardiovascular outcomes in premenopausal women, or consider the question of whether a significant interaction between age and pregnancy loss history might be observed in a younger population. Nonetheless, our findings of a strong association between a history of stillbirth and risk of incident CVD within 5 years in women aged 50–59 may support the suggestion that pregnancy loss history could be efficacious as a clinical predictor of CVD risk in younger women.

Strengths of our analysis include the large sample size, lengthy follow-up, and consistent well-documented methods for ascertainment of cardiovascular outcomes in the WHI OS. Additionally, our study has certain limitations. As noted in Methods, potential confounders of the association between pregnancy loss and CVD risk were identified *a priori* and included as covariates in our models. As these were assessed at entry into the WHI, when participants were past reproductive age, it is possible that some of the factors may in fact be mediators rather than confounders of the association between pregnancy loss and risk of CVD. Thus, adjusting for these covariates may have attenuated our ability to detect an association between exposure and outcome.

As history of pregnancy loss in WHI participants was based upon self-reported reproductive history data, exposure misclassification is a possible limitation of our analysis. However, analysis of self-reported pregnancy losses has demonstrated good agreement with medical records data (38), and maternal recall of pregnancy-related events has shown good reproducibility and validity, even for events that occurred several decades previously (39). Further, the degree of misclassification of pregnancy loss in our study sample is unlikely to differ among women who did and did not experience cardiovascular outcomes, and any such non-differential exposure misclassification would be expected to lead to more conservative results.

Potential misclassification of fatal coronary outcomes was an acknowledged limitation of the WHI outcome ascertainment (21). Additionally, as non-hospitalized heart failure and stroke were excluded from the WHI outcomes, we could not consider such events in our analysis. However, as with exposure misclassification, the effect of such outcome misclassification in our analysis would be unlikely to explain the observed associations, as there is no reason to suspect that misclassification of fatal outcomes or non-hospitalized events would occur differentially between women with and without a history of stillbirth.

The decision to exclude WHI participants with a self-reported history of CVD is a potential source of bias in our analysis. As our study sample by definition had to survive CVD-free until entry into the WHI cohort, we cannot exclude the possibility that our sample is biased in favor of event-free survival, particularly for subjects in the oldest age stratum. However, limiting the study sample to participants free of CVD at baseline enabled us to assess the risk of CVD in a previously healthy population, using the well-documented methods of ascertainment of CVD outcomes in the WHI cohort. Additionally, our methodology is consistent with that of a previous analysis of pregnancy loss and CVD in the WHI cohort (5).

Finally, although our analyses of cardiovascular outcomes did not demonstrate significant interactions between age and history of pregnancy loss, we lacked data on two factors that deserve further consideration: gestational age and maternal age at pregnancy loss. Recent studies of spontaneous late-second trimester pregnancy losses suggest that placental pathology plays an important role and that the etiology of later-term pregnancy loss is similar to that of stillbirth (40). Compared with losses in early pregnancy, late-term (after 12 weeks gestation) miscarriage and stillbirth both demonstrate stronger associations with the development of such clinical CVD risk factors as hypertension and type II diabetes; (41) correspondingly, late-term miscarriages are also likely to be more strongly associated with maternal future risk of CVD. As the WHI reproductive history questionnaire did not address gestational age at miscarriage, we were not able to examine this question in our analysis.

Similarly, while maternal age is strongly associated with the risk of pregnancy loss (42, 43), this is primarily due to an increased incidence of chromosomal abnormalities with increasing maternal age (27). As maternal factors account for a higher proportion of pregnancy losses in young women than in those of advanced maternal age, it is reasonable to speculate that losses experienced by young women may be more predictive of future CVD risk. As with gestational age, data on maternal age at pregnancy loss were not collected by the WHI; thus, we were unable to consider the potential importance of maternal age at loss in our analysis.

In summary, the results of the current analysis contribute to the literature on pregnancy loss and incident CVD and support the suggestion that the inclusion of history of pregnancy loss—and stillbirth in particular—in cardiovascular risk profiles may be clinically useful. Future research should strive to elucidate the common mechanisms underlying pregnancy loss and cardiovascular disease risk; additionally, the question of whether data on gestational age and maternal age at pregnancy loss might be useful clinical markers of CVD risk in women should be considered.

## Data availability statement

The data analyzed in this study is subject to the following licenses/restrictions: Women's Health Initiative datasets are made available to researchers upon approval of a paper proposal by WHI. Requests to access these datasets should be directed to helpdesk@whi.org.

## Ethics statement

The current analysis was reviewed and approved by Human Subjects Division, Office of Research, University of Washington. The WHI project was reviewed and approved by the Fred Hutchinson Cancer Research Center (Fred Hutch) IRB in accordance with the U.S. Department of Health and Human Services regulations at 45 CFR 46 (approval number: IR# 3467-EXT). WHI participants provided written informed consent for use of deidentified data for secondary analyses.

## Author contributions

CW, MK, DE, SP, and IP were responsible for the study design, data analysis and interpretation, and manuscript preparation. CK, RW, KP, and SS-J contributed to data analysis and interpretation and to manuscript revision. All authors have approved the final version of the manuscript for publication.
