# Supplementary material for: Pregnancy loss and risk of incident CVD within 5 years: Findings from the Women's Health Initiative
Source: Front Cardiovasc Med. 2023 Feb 21;10:1108286. doi: 10.3389/fcvm.2023.1108286 (PMC9989010; doi:10.3389/fcvm.2023.1108286)
Supplement: Supplementary file 1 [file Table_1.pdf]

Supplemental Table 1. Significance of multiplicative and additive interactions between age and pregnancy loss exposures in logistic regression analyses of cardiovascular disease (CVD) outcomes within 5 years, WHI participants ages 50-79

Outcome	Exposure	Mulitplicative interaction with age (continuous)	Additive interaction with age (<60 years vs. 60 and older)	
		p-value*	Relative excess risk due to interaction (RERI)	*p*-value
Total CVD	Any pregnancy loss	0.57	0.26 (-0.22, 0.74)	0.29
	Recurrent (2+) pregnancy loss	0.44	0.52 (-0.21, 1.25)	0.16
	Any stillbirth	0.85	0.78 (-0.50, 2.06)	0.23
CHD	Any pregnancy loss	0.35	0.70 (-0.21, 1.60)	0.13
	Recurrent (2+) pregnancy loss	0.31	0.93 (-0.22, 2.08)	0.11
	Any stillbirth	0.11	0.73 (-1.97, 3.42)	0.60
Heart failure	Any pregnancy loss	0.79	0.001 (-1.10, 1.10)	0.99
	Recurrent (2+) pregnancy loss	0.10	-0.16 (-2.06, 1.74)	0.87
	Any stillbirth	0.43	0.58 (-2.52, 3.68)	0.71
Stroke	Any pregnancy loss	0.45	0.60 (-0.32, 1.52)	0.20
	Recurrent (2+) pregnancy loss	0.26	-0.06 (-1.79, 1.67)	0.94
	Any stillbirth	0.43	1.81 (-0.73, 4.35)	0.16

*As determined by likelihood ratio test
