# Supplementary material for: Pregnancy loss and risk of incident CVD within 5 years: Findings from the Women's Health Initiative
Source: Front Cardiovasc Med. 2023 Feb 21;10:1108286. doi: 10.3389/fcvm.2023.1108286 (PMC9989010; doi:10.3389/fcvm.2023.1108286)
Supplement: Supplementary file 2 [file Table_2.pdf]

Supplemental Table 2. Rates of cardiovascular disease (CVD) outcome events prior to age 60, among WHI participants aged 50-59 at study entry (N=24,465)

	Total CVD		CHD		Heart failure		Stroke	
	Events	Rate*	Events	Rate*	Events	Rate*	Events	Rate*
All participants	212	1.95	69	0.64	53	0.49	22	0.20
Any history of pregnancy loss	81	2.43	24	0.72	25	0.75	6	0.18
No history of pregnancy loss	131	1.73	45	0.60	28	0.37	16	0.21
History of recurrent loss	25	2.35	4	0.38	12	1.13	3	0.28
No history of recurrent loss	187	1.90	65	0.66	41	0.42	19	0.19
History of stillbirth	12	3.36	5	1.40	6	1.69	0	---
No history of stillbirth	200	1.90	64	0.61	47	0.45	22	0.21

*Per 1000 person-years of follow-up
