# Supplementary material for: Pregnancy loss and risk of incident CVD within 5 years: Findings from the Women's Health Initiative
Source: Front Cardiovasc Med. 2023 Feb 21;10:1108286. doi: 10.3389/fcvm.2023.1108286 (PMC9989010; doi:10.3389/fcvm.2023.1108286)
Supplement: Supplementary file 3 [file Table_3.pdf]

Supplemental table 3. Association between a history of pregnancy loss and incident CVD prior to age 60, among WHI participants aged 50-59 at study entry (n=24,465)

**Exposure**	**Hazard ratio, adjusted (95% CI)***			
	CVD	*CHD*	*CHF*	*Stroke*
Any history of pregnancy loss	1.17 (0.83, 1.64)	1.04 (0.56, 1.91)	1.24 (0.63, 2.45)	0.69 (0.24, 2.00)
History of recurrent (2+) pregnancy loss	0.97 (0.59, 1.62)	0.42 (0.12, 1.41)	1.61 (0.70, 3.70)	1.18 (0.30, 4.68)
Any history of stillbirth	1.38 (0.72, 2.65)	2.42 (0.94, 6.24)	2.53 (0.96, 6.65)	---

*Adjusted for age, education, number of pregnancies, race/ethnicity, smoking status, BMI, NSES, and aspirin use. CI are not corrected for multiple comparisons.
